# Impact of Improved End-Stage Renal Disease Patient Survival on Prosthetic Valve Selection in Aortic Valve Replacement: A Nationwide Cohort Analysis

**DOI:** 10.3390/jcm15083127

**Published:** 2026-04-20

**Authors:** Kyungsub Song, Yun Jin Kim, Woo Sung Jang, YoHan Bae, Ji Eon Kim, Jae-Seung Jung, Jun Ho Lee

**Affiliations:** 1 Department of Thoracic and Cardiovascular Surgery, Keimyung University Dongsan Hospital, Keimyung University College of Medicine, Daegu 42601, Republic of Korea; chest.songks@gmail.com (K.S.); whiteuri09@gmail.com (W.S.J.); qwlswlstoo@naver.com (Y.B.); 2 Department of Pre-Medicine, College of Medicine, Hanyang University, Seoul 04763, Republic of Korea; yeun0148@hanyang.ac.kr; 3 Biostatistics Lab, Medical Research Collaborating Center, Hanyang University, Seoul 04763, Republic of Korea; 4 Department of Thoracic and Cardiovascular Surgery, Korea University Anam Hospital, Korea University College of Medicine, Seoul 02841, Republic of Korea; jieonkim82@gmail.com (J.E.K.); heartistcs@korea.ac.kr (J.-S.J.)

**Keywords:** heart valve prosthesis, aortic valve replacement, chronic kidney failure, renal dialysis

## Abstract

**Background**: Earlier studies in patients with end-stage renal dysfunction (ESRD) reported no significant difference in long-term outcomes between mechanical and tissue valves after valve surgery, largely due to the limited life expectancy of this population. As survival in patients with ESRD has improved in recent years, this study evaluated whether increased life expectancy affects long-term outcomes according to valve type in patients with ESRD undergoing aortic valve replacement (AVR) using a nationwide cohort. **Methods**: We analyzed data from the Korean National Health Insurance Service database from January 2005 to December 2021. Among 474 patients with ESRD who underwent AVR, 279 received tissue valves and 195 received mechanical valves. Propensity score matching was performed to balance baseline characteristics, yielding 99 matched patient pairs. **Results**: In the matched cohort, early mortality (within 30 days) was significantly higher in the tissue valve group (16.2% vs. 4.0%; *p* = 0.008). However, long-term survival rates at 1, 5, and 10 years did not differ significantly between the groups (all *p* > 0.05). Stratification by operative era (2005–2013 vs. 2014–2021) similarly showed no significant impact of valve type on survival despite temporal advances in care. **Conclusions**: Long-term survival and complication rates after AVR in patients with ESRD were comparable between mechanical and tissue valves across operative eras. Valve selection should be guided by shared decision-making, incorporating individual life expectancy and comorbidity profiles rather than assuming mechanical valves as the default option.

## 1. Introduction

According to the United States Renal Data System (USRDS), the incidence of end-stage renal disease (ESRD) reached 124,675 in 2016, with a prevalence of 726,331 individuals [1]. Among dialysis patients with cardiovascular comorbidities, valvular heart disease is common, occurring in 14.1% of those receiving hemodialysis (HD) and 12.2% of those undergoing peritoneal dialysis (PD) [1].

Traditionally, mechanical valves have been preferred over tissue valves for patients with ESRD undergoing aortic valve replacement (AVR), largely due to concerns about accelerated structural valve degeneration (SVD) associated with tissue prostheses in this population [2,3]. Although SVD progression is recognized to be faster in dialysis-dependent patients, the actual durability of tissue valves has remained uncertain. More recent studies, however, have consistently demonstrated minimal differences in SVD and long-term outcomes between mechanical and tissue valves, suggesting that tissue prostheses may offer survival comparable to that of mechanical valves [4,5,6,7].

Multiple studies have shown that prosthesis type has minimal impact on long-term survival among patients with ESRD [8,9,10,11]. Current guidelines therefore emphasize a patient-centered, shared decision-making process when choosing between mechanical and tissue valves, considering factors such as age, life expectancy, tolerance for anticoagulation, overall cardiac condition, and individual patient preferences [12].

The comparable long-term survival observed between valve types in patients with ESRD is largely attributed to the intrinsically limited life expectancy of this population. Many patients die before SVD becomes clinically relevant, thereby diminishing the influence of prosthesis type on outcomes [13,14]. A recent meta-analysis reported that median survival following AVR in patients with ESRD ranges from only 1.4–2.6 years [15,16,17].

Nevertheless, overall survival among patients with ESRD has gradually improved over the past several decades [18,19,20]. Foster et al. reported a 12–27% reduction in dialysis-related mortality during each 5-year interval between 1995 and 2013 across all age groups [21]. These gains have been attributed to multiple factors, including advancements in ESRD management, technological innovations in dialysis therapy, the introduction of new pharmacologic agents, broader implementation of clinical practice guidelines, and general improvements in population health [21].

Given these improvements in survival, we hypothesized that SVD may become increasingly relevant in ESRD patients who receive tissue valves for AVR, potentially leading to emerging differences in long-term outcomes and reoperation rates compared with mechanical valves. Therefore, this study sought to compare contemporary and historical outcomes of mechanical versus tissue AVR in patients with ESRD.

## 2. Materials and Methods

### 2.1. Data Sources

This study used health claims data from the National Health Insurance Service (NHIS) in South Korea (NHIS-2023-1-529). The NHIS database covers approximately 96–97% of the nation’s 50 million residents and contains detailed information on demographics; diagnoses coded using the International Classification of Diseases, 10th Revision (ICD-10); surgical and endovascular procedures; hospital identifiers; and mortality records [22].

### 2.2. Definition of Covariates

Covariates, including comorbidities and concomitant procedures, are summarized in Supplementary Table S1. Comorbidities were defined as the presence of at least two relevant diagnostic codes documented within the 3 years preceding the index date, identified using ICD-10 codes.

### 2.3. Study Outcomes

The primary outcome was all-cause mortality. Secondary outcomes included bleeding reoperation, aortic valve reintervention, cerebral infarction, intracranial hemorrhage, and gastrointestinal bleeding. The follow-up period extended from the index date to either the date of death or 31 December 2021 (the final date in the database) for patients who remained alive. Mortality was assessed at 30 days and at 1, 3, 5, and 10 years, as well as across the overall follow-up period.

### 2.4. Statistical Analysis

Continuous variables were expressed as medians with interquartile ranges (IQRs), and distributional skewness was assessed using the Kolmogorov–Smirnov test. Categorical variables were summarized as frequencies and percentages, with group comparisons performed using the chi-squared test or Fisher’s exact test, as appropriate. Survival curves were constructed using the Kaplan–Meier method, and differences between groups were evaluated with the log-rank test. Cox proportional hazards models were applied to estimate hazard ratios (HRs) and 95% confidence intervals (CIs) for clinical outcomes.

Propensity score matching (PSM) was performed to achieve balanced comparisons between the tissue valve and mechanical valve groups. Propensity scores were calculated based on age, sex, and the Charlson Comorbidity Index (CCI). One-to-one near-est-neighbor matching without replacement was conducted using a caliper width of 0.25 of the pooled standard deviation of the logit of the propensity score.

Cox proportional hazards models were constructed to provide complementary estimates of the association between valve type and clinical outcomes while accounting for baseline characteristics. Multivariable models were adjusted for covariates including age, sex, comorbidities, and concomitant procedures, and multivariable risk factor analyses for long-term survival were performed in both the total and matched cohorts. All statistical tests were two-sided, and a *p*-value < 0.05 was considered statistically significant. Analyses were performed using SAS version 9.4 (SAS Institute, Inc., Cary, NC, USA).

### 2.5. Ethical Statement

This study was approved by the Institutional Review Board (IRB) of Korea University Anam Hospital (Seoul, Republic of Korea), which waived the requirement for informed consent because de-identified patient data were used in this retrospective observational study (IRB No. 2025AN0073; approval date: 10 February 2025). All procedures were conducted in accordance with relevant guidelines and regulations.

## 3. Results

### 3.1. Study Population and Baseline Characteristics

**Figure 1:**
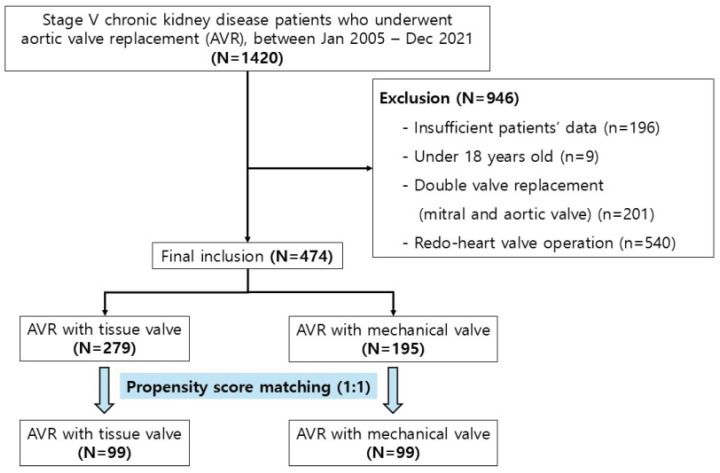
Flow diagram of patient selection for the study.

Between January 2005 and December 2021, a nationwide cohort included 1420 patients with ESRD who underwent AVR. After excluding patients with incomplete clinical data (*n* = 196), those under 18 years of age (*n* = 9), individuals undergoing double valve replacements (*n* = 201), and redo operations (*n* = 540), 474 patients remained for the final analysis. Of these, 279 (58.8%) received tissue valves, and 195 (41.2%) received mechanical valves (Figure 1). Following PSM, all standardized mean differences were reduced to below 0.1 (Supplementary Figure S1).

Baseline characteristics are detailed in Table 1. The median age of the cohort was 65.5 years (IQR, 56–72), with 38.4% female patients. The mean duration from initiation of hemodialysis to surgery was approximately 2084 days. Comorbidities, including hypertension, diabetes, chronic liver disease, and prior cerebrovascular accidents, showed some significant differences before matching; however, after PSM, both groups were well balanced, with the exception of chronic liver disease and dyslipidemia (Table 2).

### 3.2. Long-Term Survival Outcomes

In the overall cohort, mortality rates at 1, 3, 5, and 10 years were 29.3%, 37.1%, 42.6%, and 49.2%, respectively. Patients receiving mechanical valves had lower mortality rates compared with those receiving tissue valves (1-year, 22.1% vs. 34.4%; 5-year, 33.3% vs. 49.1%; 10-year, 42.6% vs. 53.8%; all *p* < 0.05) (Table 3). In the PSM-matched cohort, early mortality (within 30 days postoperatively) was 16.2% in the tissue valve group versus 4% in the mechanical valve group (*p* = 0.008). However, long-term mortality did not differ significantly between the groups. In the matched cohort, 1-, 3-, 5-, and 10-year mortality rates after AVR were 33.3%, 43.4%, 48.5%, and 52.5% in the tissue valve group, compared with 23.2%, 29.3%, 39.4%, and 50.5% in the mechanical valve group, respectively (all *p* > 0.05) (Table 4).

### 3.3. Major Complications and Adverse Events

#### 3.3.1. Aortic Valve Reoperation

In the overall cohort, reoperation rates for the aortic valve were not significantly different between the tissue and mechanical valve groups (Table 3). The 10-year reoperation rate was 3.2% in the tissue valve group and 1.5% in the mechanical valve group (*p* = 0.375). Similarly, in the PSM-matched cohort, the difference remained non-significant, with 10-year reoperation rates of 6.0% and 2.0% for the tissue and mechanical valve groups, respectively (*p* = 0.279) (Table 4).

#### 3.3.2. Cerebral Infarction, Intracranial Hemorrhage, and Gastrointestinal Bleeding

Rates of cerebral infarction, intracranial hemorrhage, and gastrointestinal bleeding did not differ significantly between the tissue and mechanical valve groups, both before and after PSM, at any time point (perioperative, 1, 3, 5, or 10 years) (Table 3 and Table 4).

In the matched cohort (Table 4), the 10-year incidence rates of cerebral infarction, intracranial hemorrhage, and gastrointestinal bleeding were 19.2% versus 20.2% (*p* > 0.999), 7.1% versus 12.1% (*p* = 0.335), and 12.1% versus 17.2% (*p* = 0.422) in the tissue and mechanical valve groups, respectively.

### 3.4. Subgroup Analyses According to Era of Operation

#### 3.4.1. Long-Term Survival in the Total Cohort

**Figure 2:**
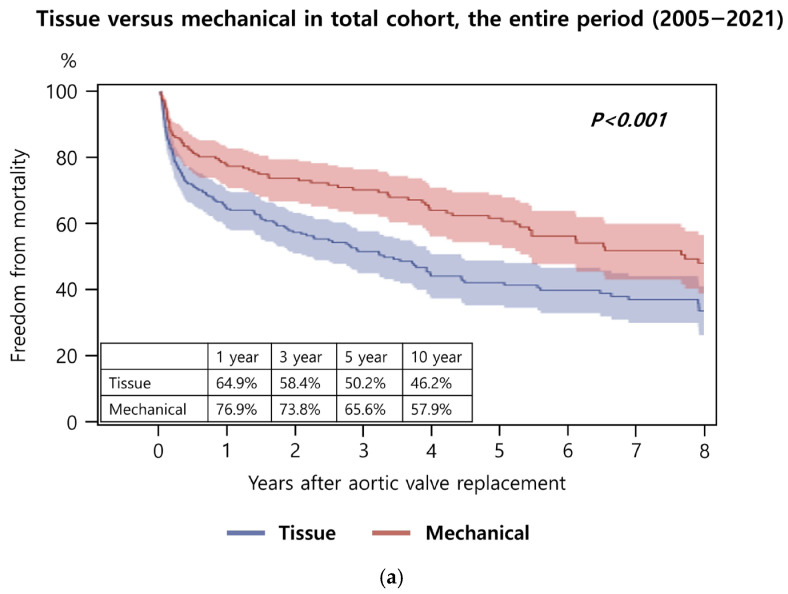

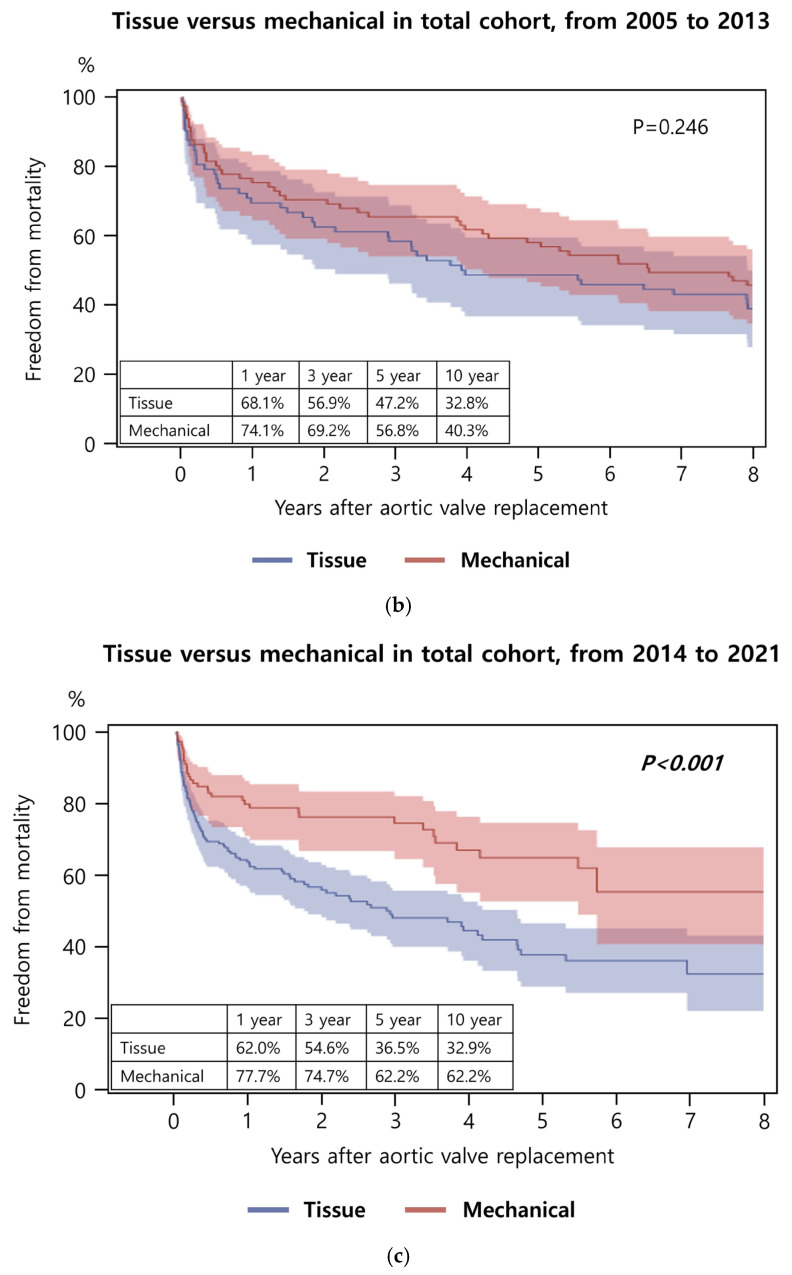

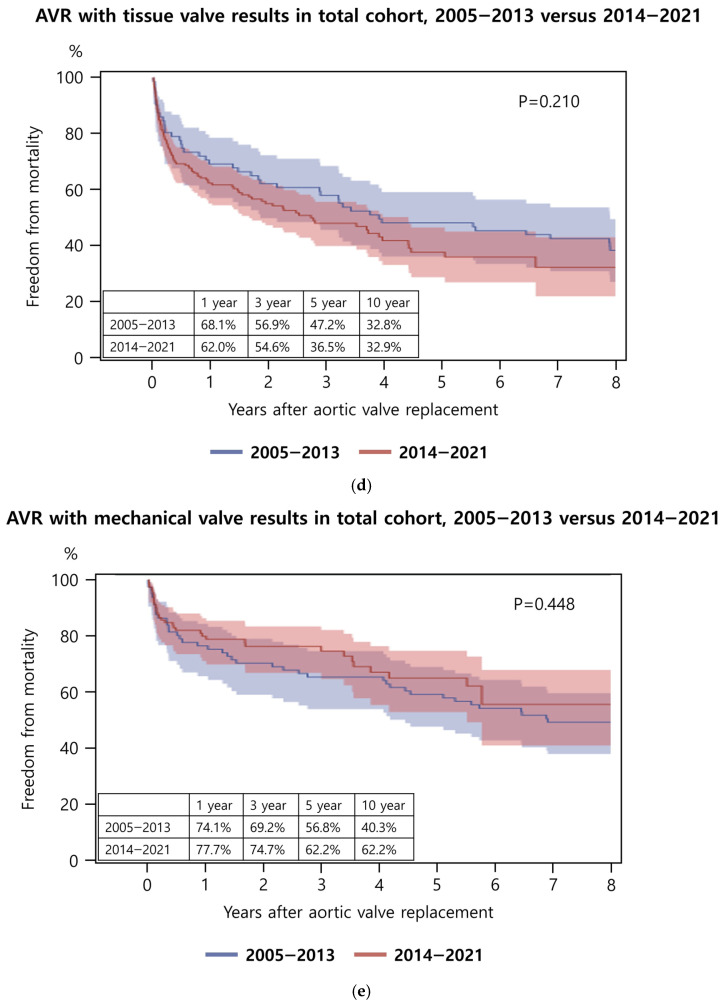
Long-term survival after aortic valve replacement (AVR) in the total cohort. (**a**) Comparison between tissue and mechanical valves in the total cohort. (**b**) Comparison between tissue and mechanical valves in patients who underwent AVR from 2005 to 2013. (**c**) Comparison between tissue and mechanical valves in patients who underwent AVR from 2014 to 2021. (**d**) Comparison of long-term survival between patients who received a tissue valve in 2005–2013 and those who received a tissue valve in 2014–2021. (**e**) Comparison of long-term survival between patients who received a mechanical valve in 2005–2013 and those who received a mechanical valve in 2014–2021.

In the overall cohort, long-term survival after AVR differed significantly between the tissue and mechanical valve groups. The 1-, 3-, 5-, and 10-year survival rates were 64.9%, 58.4%, 50.2%, and 46.2% in the tissue valve group, compared with 76.9%, 73.8%, 65.6%, and 57.9% in the mechanical valve group (*p* < 0.001) (Figure 2a). Subgroup analysis by operative era revealed that during 2005–2013, there was no significant difference in long-term survival between valve types (*p* = 0.246) (Figure 2b). In contrast, for the 2014–2021 period, patients receiving mechanical valves demonstrated superior survival compared with those receiving tissue valves (*p* < 0.001) (Figure 2c). No significant differences were observed when comparing tissue valve outcomes between the 2005–2013 and 2014–2021 periods (*p* = 0.210) (Figure 2d), nor between the mechanical valve outcomes across the same periods (*p* = 0.448) (Figure 2e).

#### 3.4.2. Long-Term Survival in the Matched Cohort

**Figure 3:**
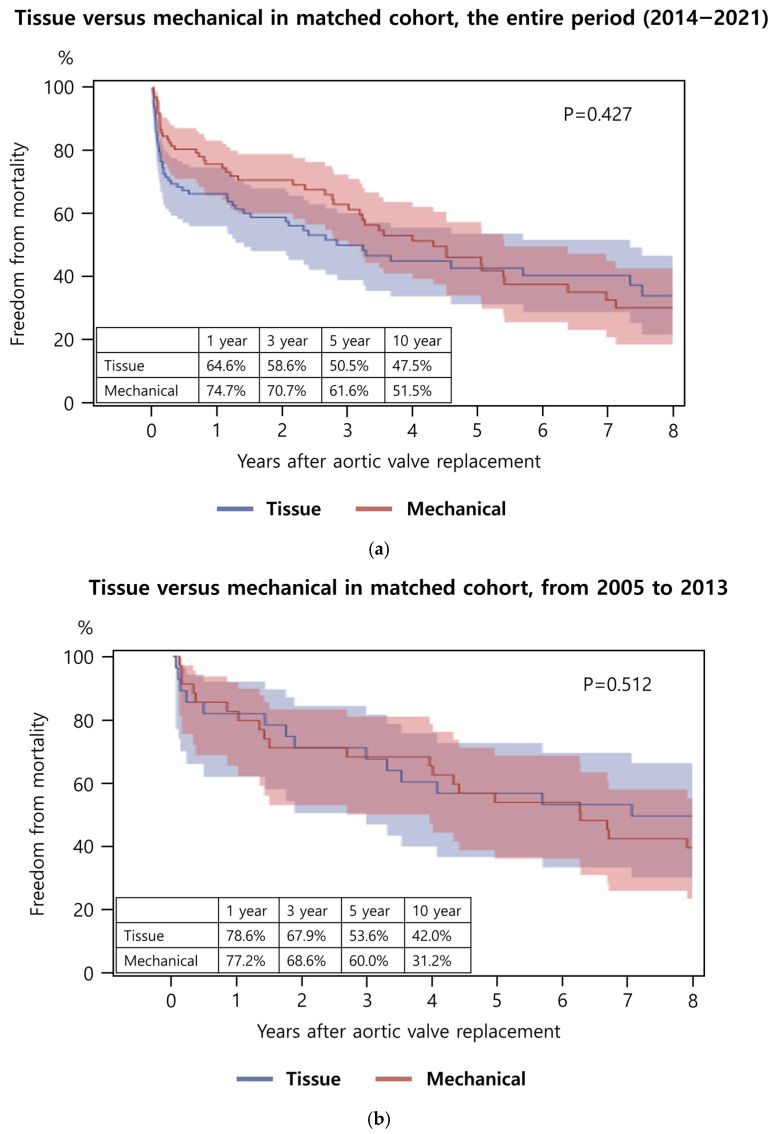

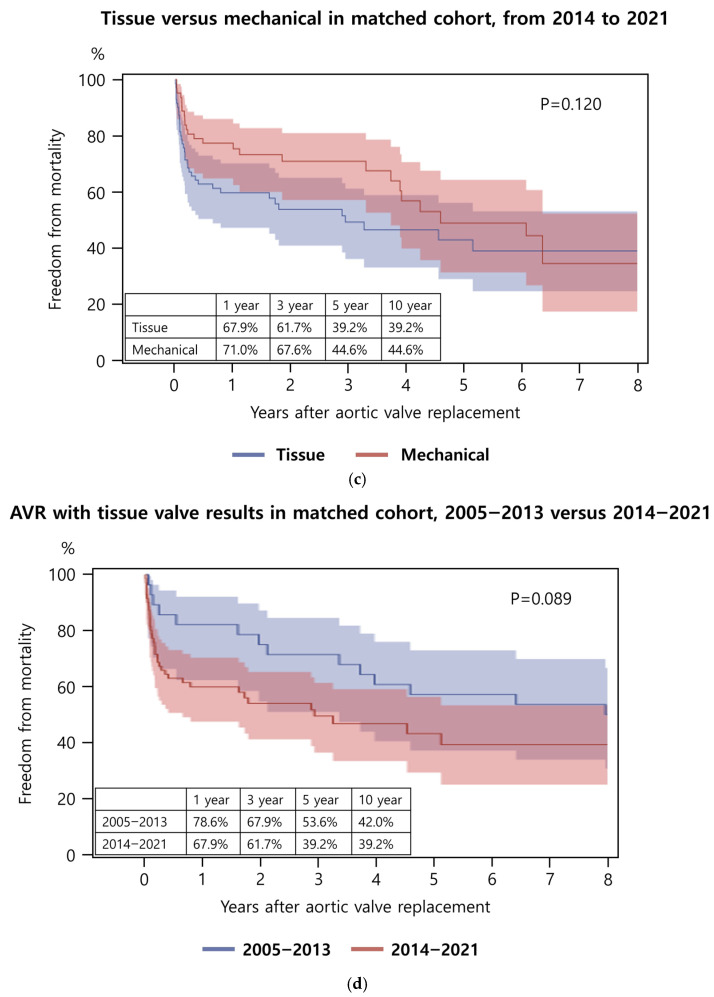

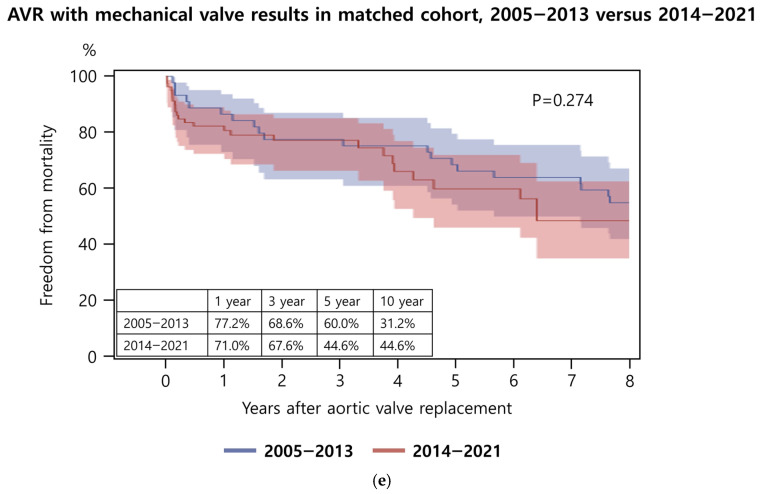
Long-term survival after aortic valve replacement (AVR) in the propensity score-matched cohort. (**a**) Comparison between tissue and mechanical valves in the total cohort. (**b**) Comparison between tissue and mechanical valves in patients who underwent AVR from 2005 to 2013. (**c**) Comparison between tissue and mechanical valves in patients who underwent AVR from 2014 to 2021. (**d**) Comparison of long-term survival between patients who received a tissue valve in 2005–2013 and those who received a tissue valve in 2014–2021. (**e**) Comparison of long-term survival between patients who received a mechanical valve in 2005–2013 and those who received a mechanical valve in 2014–2021.

After PSM, 1-, 3-, 5-, and 10-year survival rates were 64.6%, 58.6%, 50.5%, and 47.5% in the tissue valve group and 74.7%, 70.7%, 61.6%, and 51.5% in the mechanical valve group (*p* = 0.427) (Figure 3a). When analyzed by operative era, no significant differences in survival were observed between tissue and mechanical valve groups for either 2005–2013 (*p* = 0.512, Figure 3b) or 2014–2021 (*p* = 0.120, Figure 3c). Similarly, comparisons of tissue valve outcomes between the 2005–2013 and 2014–2021 periods (*p* = 0.089, Figure 3d) and mechanical valve outcomes across the same periods (*p* = 0.274, Figure 3e) showed no significant differences.

Multivariable risk factor analysis for long-term survival in both the overall and matched cohorts indicated that valve type (mechanical versus bioprosthetic) was not a significant predictor of survival in either operative era (2005–2013 or 2014–2021) (Table 5).

## 4. Discussion

This study evaluated whether improvements in patient management and the resulting increases in survival among ESRD patients have influenced outcomes according to valve type in those undergoing AVR. Our findings indicate that overall survival among patients with ESRD has improved over time compared with historical data [1,23]. However, long-term survival after AVR remained comparable between patients receiving mechanical and tissue valves, with no statistically significant differences observed between prosthesis types [9]. These results support a patient-centered approach that considers age, anticoagulant tolerance, comorbidities, and anticipated life expectancy, rather than defaulting to valve type as the primary determinant. Observations from the most recent era (2014–2021) further confirm that advancements in surgical and perioperative care have not altered the neutral effect of valve type on long-term outcomes in this population. This study provides robust evidence from a nationwide cohort of stage V chronic kidney disease patients, demonstrating the equivalence of valve types for long-term management and supporting clinical decision-making and individualized risk–benefit assessment in complex cases.

The findings of this study show no significant differences in long-term survival between mechanical and tissue valve groups among patients undergoing AVR across the entire study period (2005–2021), the earlier era (2005–2013), or the recent era (2014–2021). For temporal analyses, we used 2014 as a pragmatic boundary to distinguish earlier and more contemporary treatment periods within the study interval while maintaining adequate sample size in both groups. This cutoff was intended to facilitate temporal stratification rather than to indicate a single discrete change in clinical practice beginning in 2014. Specifically, long-term survival did not differ significantly between patients receiving tissue valves in 2005–2013 and those in 2014–2021 (*p* = 0.089). Similarly, among patients with mechanical valves, there was no significant difference in survival between the 2005–2013 and 2014–2021 periods (*p* = 0.274). These results suggest that, despite improvements in overall survival among patients with ESRD over time [23,24], valve type has not had a meaningful impact on patient survival. Furthermore, risk factor analysis for long-term survival, cerebral infarction, bleeding complications, and aortic valve reintervention showed no significant associations with prosthesis type, and complication rates remained stable across the different eras.

An unexpected finding in the matched cohort was the significantly lower 30-day mortality observed in the mechanical valve group. This result should be interpreted with caution, as it is unlikely that prosthesis type alone directly influences early postoperative mortality. Despite matching for age and overall comorbidity burden, patients selected for tissue valves may have had higher frailty, greater clinical urgency, or other perioperative risks that were not captured in the administrative claims database. Therefore, this early mortality difference may reflect residual confounding or surgeon/patient selection factors rather than a direct effect of valve prosthesis type.

Renal dysfunction is closely associated with cardiovascular disease and heart failure, forming a complex cardiorenal interaction that significantly influences outcomes in patients with valvular heart disease. In patients with ESRD, chronic volume overload, metabolic disturbances, and systemic inflammation contribute to structural and functional cardiac remodeling. These pathophysiological processes may accelerate valvular calcification and worsen cardiac performance, thereby increasing the overall cardiovascular risk profile in this population [25,26]. Particularly as heart failure progresses, symptoms such as dyspnea serve as critical prognostic indicators, being strongly associated with adverse cardiovascular outcomes [27]. Furthermore, after heart valve replacement surgery, dialysis is a major contributor to SVD following cardiac valve replacement, with valvular calcification serving as the primary mechanism driving SVD [28,29]. Among patients with at least moderate chronic kidney disease, each 1 mg/dL increase in serum phosphate is associated with a 25% and 62% higher prevalence of aortic and mitral valve calcification, respectively [30]. For this reason, earlier guidelines from the American College of Cardiology and American Heart Association recommended the use of mechanical valves in ESRD patients requiring dialysis [3]. However, recent studies have reported no significant differences in long-term outcomes between mechanical and tissue valves [4,11,31,32], although some research suggests that mechanical valves may provide superior long-term survival, particularly in AVR patients compared with those undergoing mitral valve replacement [2,14,33]. Consequently, current guidelines [12] emphasize that valve selection in patients with ESRD should be individualized, taking into account anticipated life expectancy, risks associated with anticoagulant therapy, potential for SVD, and shared decision-making. In particular, younger, otherwise healthy patients without New York Heart Association class III or IV symptoms are likely to survive long enough after valve replacement to warrant consideration of a mechanical valve [9,34].

With the increasing adoption of transcatheter aortic valve replacement, the management landscape of aortic valve disease continues to evolve. In particular, valve-in-valve procedures have emerged as a potential strategy for managing structural valve degeneration in patients who initially receive bioprosthetic valves. Although our dataset did not allow evaluation of transcatheter interventions, these evolving treatment options may further influence prosthesis selection strategies in patients with ESRD.

Another notable finding was the lack of a significant difference in bleeding outcomes between the mechanical and tissue valve groups. Although mechanical prostheses are generally associated with increased bleeding risk due to lifelong anticoagulation therapy, this pattern was not observed in our cohort. This may partly reflect the intrinsically elevated bleeding risk in patients with ESRD. Moreover, the administrative claims database used in this study does not capture detailed information on anticoagulation intensity, international normalized ratio control, or minor bleeding events. Accordingly, the similar bleeding outcomes observed between groups likely reflect both the limitations of the dataset and the high baseline bleeding risk in this population, rather than a true absence of bleeding risk associated with mechanical valves.

### Limitations

This study has several limitations. First, because the analysis relied on a nationwide administrative database, detailed clinical variables that may influence outcomes after AVR, such as frailty, hemodynamic status, operative urgency, and detailed echocardiographic findings, were not available. Second, patient-specific clinical considerations influencing prosthesis selection, including surgeon preference, anatomical characteristics, and individualized perioperative risk assessment, could not be fully captured in the claims data. Third, although long-term outcomes and complications were analyzed, the use of insurance claims data may have resulted in incomplete ascertainment of certain clinical events and did not provide detailed information on anticoagulation intensity, international normalized ratio control, or minor bleeding episodes. Fourth, the retrospective observational design introduces the possibility of residual confounding despite the use of propensity score matching. Fifth, the substantial reduction in sample size after PSM may have preferentially selected patients with greater baseline overlap. Accordingly, the propensity score-matched results should be interpreted as findings from a selected subgroup with adequate baseline overlap, rather than as estimates fully generalizable to the entire nationwide ESRD population undergoing AVR. Finally, the inclusion of patients with active malignancy may have acted as a potential confounder, as the independent prognostic impact of cancer-related mortality could have influenced the long-term survival curves.

## 5. Conclusions

Despite improvements in overall survival among patients with ESRD in recent years, this study did not observe a statistically significant difference in long-term outcomes following AVR between mechanical and tissue valves. These findings suggest that valve selection should be guided by careful consideration of the patient’s overall health, comorbidities, and anticipated life expectancy, followed by shared decision-making between the patient and physician.

## Figures and Tables

**Table 1:** Baseline characteristics of patients in the total cohort.

	Total (*n* = 474)	Tissue Valve (*n* = 279)	Mechanical (*n* = 195)	*p*-Value
Age (years), median (IQR)	65.5 (56–72)	70 (65–75)	56 (49–62)	***<0.001***
Age group, *n* (%)				***<0.001***
<50	101 (21.3)	16 (5.7)	85 (43.6)	
55–64	121 (25.5)	46 (16.5)	75 (38.5)	
≥65	252 (53.2)	217 (77.8)	35 (18.0)	
Sex, female, *n* (%)	182 (38.4)	115 (41.2)	67 (34.4)	0.150
Time to surgery from hemodialysis, days, median (IQR)	2084 (505–3631)	2075 (391–3441)	2104 (776–3695)	0.398
Year of surgery, *n* (%)				***<0.001***
2005–2013	153 (32.3)	72 (25.8)	81 (41.5)	
2014–2021	321 (67.7)	207 (74.2)	114 (58.5)	
CCI, *n* (%)				***0.049***
0	3 (0.6)	2 (0.7)	1 (0.5)	
1	7 (1.5)	3 (1.1)	4 (2.1)	
2	15 (3.2)	5 (1.8)	10 (5.1)	
3–4	34 (7.2)	15 (5.4)	19 (9.7)	
5	415 (87.6)	254 (91.0)	161 (82.6)	
CCI, median (IQR)	9 (7–12)	10 (7–13)	9 (6–11)	***0.001***
Comorbidities, *n* (%)				
Hypertension	450 (94.9)	269 (96.4)	181 (92.8)	0.091
Diabetes mellitus	374 (78.9)	228 (81.7)	146 (74.9)	0.086
Chronic liver disease	37 (7.2)	27 (9.7)	7 (3.6)	***0.011***
Dyslipidemia	418 (88.2)	245 (87.8)	173 (88.7)	0.885
Chronic obstructive pulmonary disease	87 (18.4)	67 (24.0)	20 (10.3)	***<0.001***
Cancer	124 (26.2)	88 (31.5)	36 (108.5)	***0.001***
Cerebral infarction	138 (29.1)	93 (33.3)	45 (23.1)	***0.018***
Congestive heart failure	265 (55.9)	163 (58.4)	102 (52.3)	0.190
Arrhythmia	116 (24.5)	85 (30.5)	31 (15.9)	***<0.001***
Atrial fibrillation	108 (22.8)	68 (24.4)	40 (20.5)	0.374
Myocardial infarction	41 (8.7)	28 (10.0)	13 (6.7)	0.246
Concomitant procedures, *n* (%)				
Tricuspid valve surgery	46 (9.7)	25 (9.0)	21 (10.8)	0.531
Mitral valve surgery	33 (7.0)	16 (5.7)	17 (8.7)	0.271
Arrhythmia surgery	25 (5.3)	18 (6.5)	7 (3.6)	0.212
Aorta surgery	66 (13.9)	41 (14.7)	25 (12.8)	0.592
Coronary artery bypass grafting	90 (19.0)	65 (23.3)	25 (12.8)	***0.004***

Data are presented as numbers (%) or medians (interquartile ranges). Significant *p*-values are shown in italics and bold. CCI, Charlson Comorbidity Index.

**Table 2:** Baseline characteristics of patients after propensity score matching.

	Total (*n* = 198)	Tissue Valve (*n* = 99)	Mechanical (*n* = 99)	*p*-Value
Age (years), median (IQR)	63.0 (58–66)	63.0 (59–66)	62.0 (57–66)	0.277
Age group, *n* (%)				0.659
<50	32 (16.1)	14 (14.1)	18 (18.1)	
55–64	91 (46.0)	45 (45.5)	46 (46.5)	
≥65	75 (37.9)	40 (40.4)	35 (35.4)	
Sex, female, *n* (%)	90 (45.5)	45 (45.5)	45 (45.5)	>0.999
Time to surgery from hemodialysis, days, median (IQR)	2317 (720–3695)	2322 (425–3994)	2201.5 (769–3602)	0.788
Year of surgery, *n* (%)				0.081
2005–2013	63 (31.8)	28 (28.3)	35 (35.4)	
2014–2021	135 (68.2)	71 (71.7)	64 (64.6)	
CCI, *n* (%)				>0.999
0	2 (1.0)	1 (1.0)	1 (1.0)	
1	1 (0.5)	0	1 (1.0)	
2	1 (0.5)	1 (1.0)	0	
3–4	12 (6.1)	6 (6.1)	6 (6.1)	
5	182 (91.9)	91 (91.9)	91 (91.9)	
CCI, median (IQR)	10 (7–12)	10 (7–12)	9 (7–11)	0.544
Comorbidities, *n* (%)				
Hypertension	192 (97.0)	96 (97.0)	96 (97.0)	>0.999
Diabetes mellitus	162 (81.8)	79 (79.8)	83 (83.8)	0.581
Chronic liver disease	20 (10.1)	17 (17.2)	3 (3.0)	***0.002***
Dyslipidemia	184 (92.9)	88 (88.9)	96 (97.0)	***0.049***
Chronic obstructive pulmonary disease	33 (16.7)	22 (22.2)	11 (11.1)	0.055
Cancer	57 (28.8)	35 (35.4)	22 (22.2)	0.059
Cerebral infarction	64 (32.3)	37 (37.4)	27 (27.3)	0.171
Congestive heart failure	111 (56.1)	57 (57.6)	54 (54.6)	0.775
Arrhythmia	54 (27.3)	32 (32.3)	22 (22.2)	0.151
Atrial fibrillation	40 (20.2)	22 (22.2)	18 (18.2)	0.596
Myocardial infarction	19 (9.6)	10 (10.1)	9 (9.1)	>0.999
Concomitant procedures, *n* (%)				
Tricuspid valve surgery	19 (9.6)	9 (9.1)	10 (10.1)	>0.999
Mitral valve surgery	15 (7.6)	5 (5.1)	10 (10.1)	0.283
Arrhythmia surgery	8 (4.0)	4 (4.0)	4 (4.0)	>0.999
Aorta surgery	25 (12.6)	15 (15.2)	10 (10.1)	0.393
Coronary artery bypass grafting	30 (15.2)	19 (19.2)	11 (11.1)	0.165

Data are presented as numbers (%) or medians (interquartile ranges). Significant *p*-values are shown in italics and bold. CCI, Charlson Comorbidity Index.

**Table 3:** Unadjusted long-term clinical outcomes in the total cohort.

	Total (*n* = 474)	Tissue (*n* = 279)	Mechanical (*n* = 195)	*p*-Value
Mortality, *n* (%)				
Within 30 days	44 (9.3)	34 (12.2)	10 (5.3)	***0.010***
1-year	139 (29.3)	96 (34.4)	43 (22.1)	***0.004***
3-year	176 (37.1)	122 (43.7)	54 (27.7)	***0.001***
5-year	202 (42.6)	137 (49.1)	65 (33.3)	***0.001***
10-year	233 (49.2)	150 (53.8)	83 (42.6)	***0.020***
Overall	239 (50.4)	154 (55.2)	85 (43.6)	***0.015***
Bleeding reoperation	71 (15.0)	44 (15.8)	27 (13.9)	0.6030
Aortic valve reintervention, *n* (%)				
1-year	3 (0.6)	2 (0.7)	1 (0.5)	>0.999
3-year	7 (1.5)	5 (1.8)	2 (1.0)	0.705
5-year	8 (1.7)	6 (2.2)	2 (1.0)	0.480
10-year	12 (2.5)	9 (3.2)	3 (1.5)	0.375
Overall	13 (2.7)	10 (3.6)	3 (1.5)	0.255
Cerebral infarction, *n* (%)				
During hospitalization	25 (5.3)	12 (4.3)	13 (6.7)	0.299
1-year	58 (12.2)	33 (11.8)	25 (12.8)	0.777
3-year	71 (15.0)	40 (14.3)	31 (15.9)	0.695
5-year	84 (17.7)	48 (17.2)	36 (18.5)	0.807
10-year	88 (18.6)	51 (18.3)	37 (19.0)	0.905
Overall	89 (18.8)	52 (18.6)	37 (19.0)	>0.999
Intracranial hemorrhage, *n* (%)				
During hospitalization	0	0	0	-
1-year	18 (3.8)	13 (4.7)	5 (2.6)	0.330
3-year	29 (6.1)	20 (7.2)	9 (4.6)	0.331
5-year	36 (7.6)	20 (7.2)	16 (8.2)	0.726
10-year	45 (9.5)	23 (8.2)	22 (11.3)	0.270
Overall	46 (9.7)	23 (8.2)	23 (11.8)	0.210
Gastrointestinal bleeding, *n* (%)				
During hospitalization	0	0	0	-
1-year	29 (6.1)	19 (6.8)	10 (5.1)	0.560
3-year	39 (8.2)	28 (10.0)	11 (5.6)	0.092
5-year	48 (10.1)	32 (11.5)	16 (8.2)	0.281
10-year	59 (12.5)	34 (12.2)	25 (12.8)	0.888
Overall	61 (12.9)	34 (12.2)	27 (13.9)	0.676

Data are presented as numbers (%). Significant *p*-values are shown in italics and bold.

**Table 4:** Long-term clinical outcomes after propensity score matching.

	Total (*n* = 198)	Tissue (*n* = 99)	Mechanical (*n* = 99)	*p*-Value
Mortality, *n* (%)				
Within 30 days	20 (10.1)	16 (16.2)	4 (4.0)	***0.008***
1-year	56 (28.3)	33 (33.3)	23 (23.2)	0.155
3-year	72 (36.4)	43 (43.4)	29 (29.3)	0.054
5-year	87 (43.9)	48 (48.5)	39 (39.4)	0.252
10-year	102 (51.5)	52 (52.5)	50 (50.5)	0.887
Overall	105 (53.0)	54 (54.6)	51 (51.5)	0.776
Bleeding reoperation	25 (12.6)	10 (10.1)	15 (15.2)	0.393
Aortic valve reintervention, *n* (%)				
1-year	1 (0.5)	0	1 (1.0)	>0.999
3-year	5 (2.5)	3 (3.0)	2 (2.0)	>0.999
5-year	6 (3.0)	4 (4.0)	2 (2.0)	0.683
10-year	8 (4.0)	6 (6.0)	2 (2.0)	0.279
Overall	9 (4.6)	7 (7.1)	2 (2.0)	0.170
Cerebral infarction, *n* (%)				
During hospitalization	10 (5.1)	2 (2.0)	8 (8.1)	0.101
1-year	24 (12.1)	11 (11.1)	1 3 (13.1)	0.828
3-year	30 (15.2)	15 (15.2)	15 (15.2)	>0.999
5-year	36 (18.2)	1 7(17.2)	19 (19.2)	0.854
10-year	39 (19.7)	19 (19.2)	20 (20.2)	>0.999
Overall	39 (19.7)	19 (19.2)	20 (20.2)	>0.999
Intracranial hemorrhage, *n* (%)				
During hospitalization	0	0	0	
1-year	6 (3.0)	3 (3.0)	3 (3.0)	>0.999
3-year	9 (4.6)	5 (5.1)	4 (4.0)	>0.999
5-year	15 (7.6)	5 (5.1)	10 (10.1)	0.283
10-year	19 (9.6)	7 (7.1)	12 (12.1)	0.335
Overall	20 (10.1)	7 (7.1)	13 (13.1)	0.238
Gastrointestinal bleeding, *n* (%)				
During hospitalization	0	0	0	
1-year	12 (6.1)	5 (5.1)	7 (7.1)	0.767
3-year	17 (8.6)	9 (9.1)	8 (8.1)	>0.999
5-year	23 (11.6)	11 (11.1)	12 (12.1)	>0.999
10-year	29 (14.7)	12 (12.1)	17 (17.2)	0.422
Overall	31 (15.7)	12 (12.1)	19 (19.2)	0.240

Data are presented as numbers (%). Significant *p*-values are shown in italics and bold.

**Table 5:** Comparative outcomes of mechanical vs. bioprosthetic aortic valve replacement using multivariable risk factor analysis, according to era of operation.

	All Study Patients		Propensity Score-Matched Patients	
	Adjusted HR (95% CI)	*p*-Value	HR (95% CI)	*p*-Value
**Between 2005 and 2013**				
Mortality				
1-year	0.81 (0.44–1.51)	0.509	0.79 (0.02–28.19)	0.894
3-year	0.77 (0.42–1.39)	0.378	1.12 (0.16–7.76)	0.908
5-year	0.90 (0.52–1.55)	0.702	1.01 (0.25–4.09)	0.990
10-year	1.00 (0.62–1.61)	0.983	1.16 (0.47–2.85)	0.754
Overall	0.96 (0.60–1.53)	0.849	1.07 (0.50–2.29)	0.854
Bleeding reoperation	0.50 (0.18–1.39)	0.495	22.25 (0.18–2737.58)	0.206
**Between 2014 and 2021**				
Mortality				
1-year	0.61 (0.30–1.24)	0.174	0.65 (0.29–1.46)	0.299
3-year	0.57 (0.29–1.12)	0.102	0.70 (0.33–1.50)	0.356
5-year	0.64 (0.35–1.18)	0.154	0.89 (0.44–1.75)	0.711
10-year	0.69 (0.38–1.25)	0.221	0.97 (0.50–1.90)	0.928
Overall	0.69 (0.38–1.25)	0.221	0.97 (0.50–1.90)	0.928
Bleeding reoperation	0.53 (0.18–1.60)	0.262	0.56 (0.11–2.83)	0.480

## Data Availability

The data presented in this study are available on request from the corresponding author. The data are not publicly available due to privacy restrictions.

## Supplementary Materials

The following supporting information can be downloaded at: https://www.mdpi.com/article/10.3390/jcm15083127/s1, Figure S1. Distribution of the Standardized Mean Difference before and after propensity score matching. Table S1. The main procedure, comorbidities, and concomitant procedure codes.

## Abbreviations

The following abbreviations are used in this manuscript:

ACC/AHA	American College of Cardiology/American Heart Association
AVR	Aortic valve replacement
CCI	Charlson Comorbidity Index
CI	Confidence interval
ESRD	End-stage renal disease
HD	Hemodialysis
HR	Hazard ratio
ICD-10	International Classification of Diseases, 10th Revision
IQR	Interquartile range
IRB	Institutional Review Board
NHIS	National Health Insurance Service
NYHA	New York Heart Association
PD	Peritoneal dialysis
PSM	Propensity score matching
SVD	Structural valve degeneration
USRDS	United States Renal Data System
