# Supplementary material for: Impact of Improved End-Stage Renal Disease Patient Survival on Prosthetic Valve Selection in Aortic Valve Replacement: A Nationwide Cohort Analysis
Source: J Clin Med. 2026 Apr 20;15(8):3127. doi: 10.3390/jcm15083127 (PMC13117633; doi:10.3390/jcm15083127)
Supplement: Supplementary file 1 [file jcm-15-03127-s001.zip › jcm-4151981-supplementary.pdf]

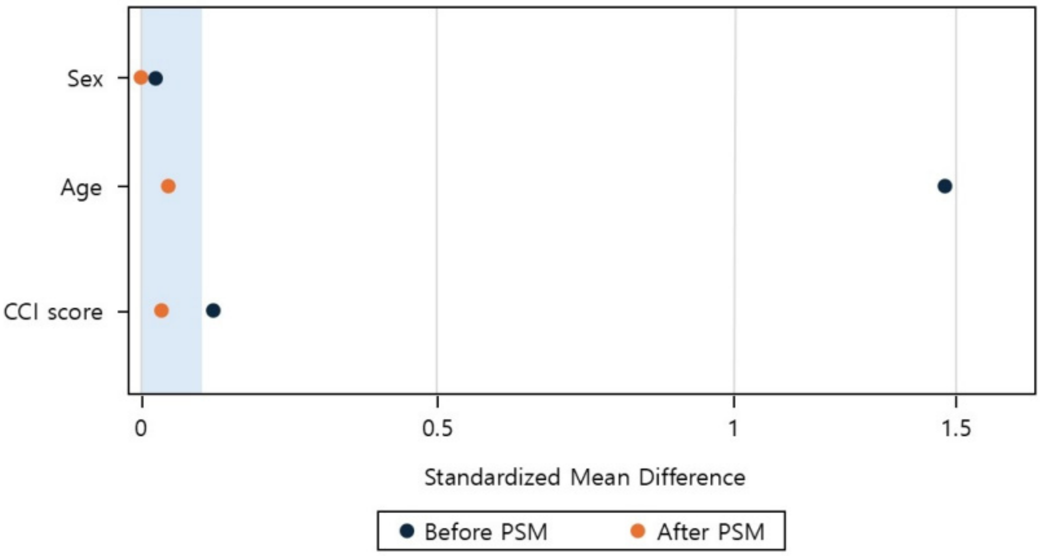

Supplementary Figure S1. Distribution of the Standardized Mean Difference before and after propensity score matching.

Supplementary Table S1. The main procedure, comorbidities, and concomitant procedure codes.

**Aortic valve replacement**	**O1793**
**Chronic kidney disease**	I12.0, I12.9, I13.1, I13.2, N17.x, N18.x, N19.x, Z49.x, Z94.0, Z99.2
**Comorbidities[a]**	
Hypertension	I10.x-I13.x, I15.x
Diabetes mellitus	E10.x-E14.x
Chronic liver disease	K70.2, K70.3, K74.x
Dyslipidemia	E78.x
Chronic obstructive pulmonary disease	J44.x
Cancer	C00.x-C99.x
Cerebral infarction	I63.x, I64.x
Congestive heart failure	I11.x, I13.0, I13.2, I15.x, I50.x, J81.x
Arrhythmia	I44.x, I45.x, I47.1, I47.9, I49.x
Atrial fibrillation	I48.x
Myocardial infarction	I21.x, I22.x
**Concomitant procedures**	
Tricuspid valve surgery	O1781, O1791, O1794
Mitral valve surgery	O1782, O1792, O1795
Arrhythmia surgery	O2006
Aorta surgery	O2031, O2032, O2033, O2034
Coronary artery bypass grafting	OA640, OA641, OA642, OA647, OA648, OA649<br>O1640, O1641, O1642, O1647, O1648, O1649

[a]Comorbidities were defined as the presence of at least two relevant diagnostic codes recorded within the three years preceding the index date, as identified using International Classification of Disease 10th Revision codes.
